# S210. MULTIDISCIPLINARY LIFESTYLE-ENHANCING TREATMENT FOR INPATIENTS WITH SEVERE MENTAL ILLNESS (MULTI-STUDY): EFFECTS ON PHYSICAL HEALTH, PSYCHOTIC SYMPTOMS, QUALITY OF LIFE AND FUNCTIONING

**DOI:** 10.1093/schbul/sby018.997

**Published:** 2018-04-01

**Authors:** Jeroen Deenik, Diederik Tenback, Ingrid Hendriksen, Erwin Tak, Peter van Harten

**Affiliations:** 1 GGz Centraal, Amersfoort; 2 LivIng Active, Santpoort-Zuid; 3 Tak advies en onderzoek, Leiden

## Abstract

**Background:**

Patients with severe mental illness (SMI) are characterized by an unhealthy lifestyle, which contributes to the widening mortality gap with the general population [1] Changing high levels of sedentary behaviour (SB) and lack of physical activity (PA) is very challenging [2–4].

Effective interventions improving lifestyle in inpatients are still limited, while of all patients with SMI, the hospitalized do have the worst health status.

We implemented a MUltidisciplinary Lifestyle Enhancing Treatment for Inpatients with SMI (MULTI), mainly including a daily structure, tailored sports- and work-related activities, attention to dietary habits, psycho-education and participation of staff. It involved a culture change which was implemented based on a ‘change-from-within’-principle, using multidisciplinary* cooperation within the current context and resources of inpatient mental healthcare.

* Psychiatrists, activity coordinators, nurse practitioners, dietician and nurses, some of them trained as lifestyle coach.

**Aim:**

Evaluate changes in physical and mental health and functioning after 18 months compared to treatment as usual (TAU).

**Methods:**

Observational controlled design including long-term hospitalised inpatients with SMI. We used data from routine screening and a previous cross-sectional study (2013), supplemented by a repeated accelerometer measurement (2015). Patients were included if they received no other intervention related to lifestyle within 18 months after the start of MULTI and if baseline accelerometer data was available. Patients were excluded from analysis if they had a lack of data after 18 months because they (1) were deceased, (2) moved or were discharged from the hospital or (3) had insufficient follow-up accelerometer data.

Measures:

Accelerometer-measured physical activity (PA) [ActiGraph GT3X+]

Metabolic health [weight, abdominal girth, blood-pressure and -levels and metabolic syndrome criteria]

Psychotic symptoms [PANSS-r]

Quality of life (QoL) [EQ-5D & WHOQoL-Bref]

Psychosocial functioning [HoNOS]

**Analysis:**

hierarchical multilevel regression using change-scores, correcting for baseline outcome-value, age, diagnosis and baseline illness-severity.

**Results:**

We had sufficient data of 65 patients receiving MULTI and 49 within TAU.

Significant (p < 0.05) improvements in total PA (B = 0.5), moderate-to-vigorous PA (B = 1.8%), weight (B = -4.2kg), abdominal girth (B = -3.5cm), systolic blood-pressure (B = -8.0mmHg), HDL-cholesterol (B = 0.1mmol/l) and psychosocial functioning on sums score (B = -3.6), impairment (B = -0.7) and social problems (B = -3.0). No improvements were observed in PA/metabolic health within TAU. Patients receiving MULTI had higher odds to recover from ≥1 metabolic syndrome criterion (OR = 2.06). There was no significant effect on psychotic symptoms. QoL improved significantly in both groups.

**Discussion:**

Striking results for clinical practice, as much effort and attempts on lifestyle within inpatients with SMI failed to achieve desired improvements, especially in longer term.

A turnaround in inpatient mental healthcare: the negative trend of deterioration within these patients can be stopped, relevant parameters can even be positively reversed and negative effects are absent.

TAU does not improve physical health A sustainable solution towards a healthier lifestyle in inpatients with SMI at our fingertips, as MULTI was implemented using current context and resources.

